# Cardiovascular involvement and manifestations of systemic Chikungunya virus infection: A systematic review

**DOI:** 10.12688/f1000research.11078.2

**Published:** 2017-05-02

**Authors:** María Fernanda Alvarez, Adrián Bolívar-Mejía, Alfonso J. Rodriguez-Morales, Eduardo Ramirez-Vallejo

**Affiliations:** 1 Faculty of Health, Universidad Industrial de Santander, Santander, Colombia; 2 Department of Internal Medicine, Universidad Industrial de Santander, Santander, Colombia; 3 Public Health and Infection Research Group, Faculty of Health Sciences, Universidad Tecnológica de Pereira, Pereira, Risaralda, Colombia; 4 Colombian Collaborative Network on Zika and other Arboviruses (RECOLZIKA), Pereira, Risaralda, Colombia

**Keywords:** cardiovascular, Chikungunya, clinical, Colombia, Latin America

## Abstract

Background: In the last three years, chikungunya virus disease has been spreading, affecting particularly the Americas, producing more than two million cases. In this setting, not only new disease-related epidemiological patterns have been found, but also new clinical findings have been reported by different research groups. These include findings on the cardiovascular system, including clinical, electrocardiographic and echocardiographic alterations. No previous systemic reviews have been found in major databases about it.

Methods: We performed a systematic review looking for reports about cardiovascular compromise during chikungunya disease. Cardiac compromise is not so common in isolated episodes; but countries where chikungunya virus is an epidemic should be well informed about this condition. We used 6 bibliographical databases as resources: Medline/Pubmed, Embase, ScienceDirect, ClinicalKey, Ovid and SciELO. Dengue reports on cardiovascular compromise were included as well, to compare both arbovirus’ organic compromises. Articles that delved mainly into the rheumatic articular and cutaneous complications were not considered, as they were not in line with the purpose of this study. The type of articles included were reviews, meta-analyses, case-controls, cohort studies, case reports and case series. This systematic review does not reach or performed a meta-analysis.

Results: Originally based on 737 articles, our reviewed selected 40 articles with 54.2% at least mentioning CHIKV cardiovascular compromise within the systemic compromise. Cardiovascular manifestations can be considered common and have been reported in France, India, Sri Lanka, Malaysia, Colombia, Venezuela and USA, including mainly, but no limited to: hypotension, shock and circulatory collapse, Raynaud phenomenon, arrhythmias, murmurs, myocarditis, dilated cardiomyopathy, congestive insufficiency, heart failure and altered function profile (Troponins, CPK).

Conclusions: Physicians should be encouraged to keep divulgating reports on the cardiovascular involvement of chikungunya virus disease, to raise awareness and ultimately encourage suitable diagnosis and intervention worldwide. More research about cardiovascular involvement and manifestations of systemic Chikungunya virus infection is urgently needed.

## Introduction

### Rationale

Chikungunya virus (CHIKV) is an RNA-type arbovirus species that according to the International Committee on Taxonomy of Viruses (ICTV) belongs to the Togaviridae family and Alphavirus genus, along with more than 30 other pathogens for vertebrates and humans, causing a very broad spectrum of disease ^1, 2^. The word “Chikungunya” means “which contorts or bends up” in Makonde language from Tanzania and Mozambique, referring accurately to the difficulty in deambulation or walking of those affected ^1, 2^. Despite CHIKV first being documented in 1954 in Tanzania, Africa and subsequently Asia ^1, 3, 4^, it was not until 2006 that CHIKV first alarmed the world for being a major public health concern. After an explosive epidemic outbreak in French island La Réunion, where 35% of the total population was infected over six months, CHIKV arrived to central France and extended to Germany, Italy, Norway, and Switzerland ^1^. Later on, the virus hit North, Central and South America and brought with it the concept of a “self-limited febrile illness”, a more benign type of infection with predominantly articular symptomatology ^1, 3– 5^.

Alphaviruses can be separated into two phylogenetic categories: “Old World” viruses and “New World” viruses. “Old World” viruses such as CHIKV are known for their articular tropism and exanthematous febrile syndrome; and the “New World” viruses such as the western equine encephalitis and Venezuelan equine encephalitis viruses ^1– 3^ have preference for nervous system stromal cells. CHIKV infection pathway in humans is shared with Dengue fever, and is caused by the biting of borne-arthropods from the *Aedes* mosquito family, *Aedes aegypti* and most recently *Aedes albopictus* ^1^, the last one being essential to the wide geographic colonization process ever since a new mutation (A226V) in CHIKV has conferred the virus a better ability to replicate in this species. *Ae. albopictus* is more common in Asia, and has become worthy of mentioning in the Southeast of the United States and the Caribbean region ^6^. CHIKV currently circulating in America seems to no longer be related to the African lineage, but to strains documented in Asia and the Phillipines ^2, 4^.

The transmission cycle, although originally merely sylvatic between primates and forest mosquitoes, has developed an alternate urban cycle involving humans ^1, 6^. *Aedes* as vectors are capable of spreading the virus after biting a viremic human, after which CHIKV replicates in salivary glands of the female mosquito and then a new bite of a healthy host takes place ^6, 7^. After the infectious bite, the incubation period of CHIKV ranges from 1–12 days before clinical onset of symptoms ^1, 6^. The appearance of clinical manifestations of the febrile syndrome coincides with viremia settling in during a period of 5–7 days, when viral load can be as high as 10 ^9^ viral genome copies per milliliter ^3^. Most recently, cases of vertical transmission have been reported, but it is indeed rare, and transmission through nursing has not been proven ^1, 6, 8^.

Three stages of disease after the incubation period have been recognized ^9^:

• Acute (<3 weeks post-infection)
• Post-acute or subacute (3 to 12 weeks post-infection)
• Chronic (>12 weeks post-infection)

Not every patient develops the full three stages, and at least a 20% of the infected population will not develop any symptoms at all, despite serological confirmation ^3, 9^. On the other hand, isolated cases have reported severe acute manifestations, far from the classic expected evolution of the disease, especially in areas with renowned late outbreaks such as India (2006) ^1, 10^, La Réunion and Mayotte (France, 2006) ^9, 11^, Malaysia (2008), Thailand (2008) ^12, 13^ and South America (Colombia, Venezuela and later Brazil, from 2014 until now) ^14, 15^. As a result, some authors have started to classify the clinical progression of CHIKV into either classical, severe or neurological (neuro-chikungunya) ^10, 13^. The severe subtype of the disease contemplates an atypical systemic compromise, in which the liver, lungs, and even the eye are affected by the extra-articular intense inflammatory response ^10, 16, 17^. Similarly, the involvement of the heart has often been fatal and worth highlighting in some reports ^18– 22^, but it has not been very largely discussed.

Characterizing potential systemic compromise due to CHIKV infection, especially cardiovascular, and characterizing manifestations and complications as a result, is essential in clinical practice. Here, identifying the febrile syndrome is particularly common on a daily basis and, coexists in a great proportion of patients with other morbidities and chronic conditions, that could easily trigger a more severe presentation and clinical picture of the disease ^9, 11, 23^. No previous systematic reviews have been found in major databases about cardiovascular involvement and cardiovascular manifestations of chikungunya virus infection, which is the main focus of this article.

### Objectives

To systematically review published literature on the cardiovascular manifestations and involvement of systemic CHIKV infection;

• To explore which are the main clinical cardiovascular features of chikungunya infection
• To identify which are the main electrocardiographic findings of chikungunya infection

## Methods

### Protocol and registration

This protocol has followed PRISMA guidelines ( Supplementary File 1 and Supplementary File 2)

### Eligibility criteria

Any original studies that report cases with cardiovascular manifestations (acute and/or chronic) related to Chikungunya. We included studies published in English and Spanish. Eligible study designs were case-control, cohort studies, case reports and series of cases.

### Information sources

The systematic review was conducted using six bibliographical databases (Medline/Pubmed, Embase, Elsevier, ClinicalKey, Ovid and SciELO) as resources.

***Search strategy.*** To explore the extent by which this topic is currently represented in medical literature, searches were initiated with “Chikungunya AND Systemic AND Manifestations”, “Chikungunya AND Heart” and “Chikungunya AND Cardiac”. Given the lack of studies, we explored other options (such as “Chikungunya AND cardiac involvement” “Chikungunya AND cardiac complication” or “Chikungunya AND cardiovascular involvement” “Chikungunya AND cardiovascular complications” and Chikungunya AND Atypical manifestation/complications“, among others), but to be more sensitive and to include all the possibly relevant studies related to our SR, we only included studies that had been found in our initial searches. Article language was limited to English and Spanish, and there was no limit set for time of publication, but searches concluded on November 1, 2016. Dengue reports on cardiovascular compromise were included as well, to compare between both arbovirus’ organic compromise. Articles that delved mainly into the rheumatic articular and cutaneous complications were not considered, as they were not in line with the purpose of this study.

### Study selection

The type of articles included were reviews, meta-analyses, case-controls, cohort studies, case reports and case series.

### Data collection

Data extraction from reports was done independently by two investigators. They checked for duplicates and were responsible for an initial quality screening of the studies.

### Data items

During individual article assessment the variables for which data were sought included any cardiovascular manifestation associated with CHIKV infection, as well as any electrocardiographic, echocardiographic and related laboratory findings in patients during acute and/or chronic phases of Chikungunya disease.

### Risk of bias in individual studies

Chikungunya is an emerging disease in the Americas and reemerging in the world, so there are a small number of studies addressing the cardiovascular manifestations (acute and/or chronic) related to Chikungunya. The risk of bias is discussed throughout the article. To assess the quality of eligible studies critical appraisals specific to study design were completed by two independent reviewers.

We have compiled and submitted a complete review of CHIKV that includes the main facts about characterization, origin and transmission of the virus, epidemiology, pathogenesis, and clinical features of the classic and severe/atypical disease. It provides a clear focus on the extra-articular and mainly cardiovascular manifestations of the CHIKV infection, diagnosis of CHIKV-induced cardiomyopathy, management, prognosis, and differences from what can be observed with Dengue virus (DENV) infection.

## Results

### Study selection

The research initially rendered a total of 737 articles: duplicates across the databases and articles about other viruses were eliminated, unless they focused solely on cardiac compromise ( Supplementary File 2). Finally, 40 articles were selected based on their relevance and pertinence of the title or abstract to the systemic compromise that was being evaluated, with 54.2% at least mentioning CHIKV cardiovascular compromise within the systemic compromise ( Supplementary File 2).

### Study characteristics

The frequency at which the rest of the organs systems are affected is shown in Table 1. The information on the role of the cardiovascular system during CHIKV infection is very scarce indeed; only 21.4% of the resulting articles focused solely and exclusively on the cardiovascular findings; the first publication on the topic was by Obeyeskere *et al.* and dates to 1972. In relation to extra-articular compromise of other organ systems besides cardiovascular, the most published were the nervous system –both central and peripheral- and secondary skin complications.

**Table 1.** Frequency at which different organ systems are affected during CHIKV infection.

Affected organs	Reporting articles	Frequency of compromise	Countries of origin
Osteoarticular	97.1% (34)	**Extremely common**	• France, Italy • India, Sri Lanka, Malaysia, Singapore, Thailand Colombia, Venezuela, Peru • USA
**Cardiovascular**	54.2% (19)	**Common**	• France • India, Sri Lanka, Malaysia • Colombia, Venezuela • USA
**Neurological**	37.1% (13)	**Unusual**	• France • India, Singapore, Thailand • Colombia, Peru
**Skin and mucous membranes** **complications**	25.7% (9)	**Unusual**	• France • India, Sri Lanka • Colombia, Peru, Venezuela
**Renal**	22.8% (8)	**Unusual**	• France • India, Sri Lanka, Malaysia • Venezuela
**Gastrointestinal tract**	20% (7)	**Unusual**	• France • Singapore • Colombia, Peru
**Hepatic**	20% (7)	**Unusual**	• France • India, Malaysia, Singapore
**Hematological**	20% (7)	**Unusual**	• France • India, Malaysia • Colombia, Peru
**Ocular**	14.3% (5)	**Rare**	• France • India, Sri Lanka • Colombia, Peru
**Respiratory**	14.3% (5)	**Rare**	• France • India, Sri Lanka • Colombia
**Endocrine**	5.7% (2)	**Extremely rare**	• France • India

Reporting articles n () and % corresponded to the number of articles (out of the eligible n=40), that described the type of compromise (e.g. osteoarticular, cardiovascular, etc). The frequency of different organ-type of manifestations was classified as follows: extremely common (100-80%), very common (79-60%), common (59-40%), unusual (39-20%), rare (19-10%) and extremely rare if below 10%.

According to the system that is compromised (e.g. osteoarticular, cardiovascular, neurological, etc) in the literature, the frequency of compromise of organs/systems was separated into six categories: extremely common (100-80%), very common (79-60%), common (59-40%), unusual (39-20%), rare (19-10%) and extremely rare if below 10%. Data were registered in Table 1, showing the countries of origin of reports describing such types of manifestations of CHIKV infection.

***Clinical course.***The acute stage extends from the first symptomatic day to the 21 ^st^ day and is characterized by an end-of-incubation sudden high fever (often above 39°C), headaches, myalgia and the insidious onset of typical symmetric, bilateral polyarthalgia (most frequently of small distal joints – phalanges, wrists, ankles), along with a typical maculopapular evanescent rash ^1, 3, 9^. The location of the arthralgias tends to vary between individuals. There are rare descriptions in the literature of pain in the costochondral, hip and temporomandibular articulations ^24^, so it may not be advisable to dismiss a CHIKV diagnosis if these pains are present. Palmo-plantar pruritus, photophobia, edema in the face and extremities and adenopathies have been also described, and benign and self-limited hemorrhagic manifestations are relatively common in children. Subsequently, by the end of the acute stage, asthenia and adynamia tends to appear ^1, 9^.

In the post-acute stage, from the first to the third month, all symptoms described above tend to vanish, except for some residual arthralgia, and some residual fever and adynamia. Extra-articular rheumatisms such as tenosynovitis, bursitis, tendinitis, worsening of osteoarthritis and even tunnel syndrome and Raynaud phenomenon have been reported ^9^. Not every patient develops this phase, and degrees of severity and functional limitation will depend on patients’ previous comorbidities, mainly musculoskeletal. Alternatively, other risk factors for being still symptomatic after the first month have been linked, for instance, to having poor rest during the acute phase, and females above the age of 40 are at major risk ^1, 9, 11^.

Chronic CHIKV infection would be defined as a symptomatic period longer than three months and manifestations (continuous or episodic) that last for months, years or even a decade. Manifestations are the same as previously described in the post-acute phase, presenting as oscillating arthralgias over time with or without inflammatory signs until, according to natural history of the disease, the patient returns to the health state that they had before the infection. The degree of functional limitation may vary from little to moderate; leaving to a mean of 50% the most incapacitating and aggressive compromise ^9^.

***Atypical presentations.*** Atypical presentations of CHIKV infection can involve almost every organ system, as seen in Table 2. Even though the most common extra-articular manifestations reported in the literature involve the nervous system ^25– 27^ and the eye ^17^; alterations in the gastrointestinal tract, liver ^16^, kidney, muscles, mucous membranes and skin and hematologic cells have been evidenced, as well as in hemostasis and coagulation processes. Cardiovascular compromise is worthy of mentioning because of its usually fatal outcomes ^10, 28^. Infection can lead to cardiovascular manifestations, but in addition, patients with existing cardiovascular disease can deteriorate quickly, worsening the short-term prognosis; as it has been described with diabetes, lupus; or neurological, renal, pulmonary and cardiovascular insufficiency ^9, 11, 23^.

**Table 2.** Systemic extra-articular involvement of atypical CHIKV presentation ^1, 3, 6, 9– 11, 15, 16, 23– 29^. ALAT: Alanine-aminotranspherase, ASAT: Aspartate-aminotranspherate, CPK: Creatine-phosphokinase, SCr: serum creatinine, BUN: Blood Urea Nitrogen.

Affected system	Clinical manifestations
**Neurological**	• Fulminant meningoencephalitis* • Myelopathy • Encephalopathy • Polyneuropathy, optic neuritis and tunnel syndrome • Dysautonomy • Flaccid paralysis • Stroke • Cerebral edema* • Confusion and other sensory alterations • Seizures and psychomotor sequelae* • Guillain-Barre Syndrome • Cerebellar syndrome • Sensorineural hearing loss
**Skin and mucous membranes**	• Bullous dermatosis* • Skin dyschromia • Hemorrhagic lesions (gingivorrhagia, epistaxis, cutaneous)* • Oral and genital ulceration • Conjunctivitis
**Gastrointestinal tract**	• Pharyngitis • Severe abdominal pain* • Diarrhea* • Vomit* • Internal bleeding (hematemesis and melena)*
**Liver**	• Fulminant hepatitis • Hepatomegaly* • Ascites* • Altered function profile (ALAT, ASAT, bilirubin)
**Hematological**	• Thromboembolism • Intravascular coagulation • Thrombocytopenia* • Lymphopenia*
**Cardiovascular**	• Hypotension* • Shock and circulatory collapse • Raynaud phenomenon • Arrhythmias • Murmurs* • Myocarditis* • Dilated cardiomyopathy* • Congestive insufficiency* • Heart failure • Altered function profile (Troponins, CPK)
**Respiratory**	• Dyspnea • Pulmonary edema • Pneumonia • Pleural effusion
**Renal**	• Albuminuria • Hematuria • Nephritis • Acute renal failure* • Altered function profile (SCr, BUN)
**Ocular**	• Uveitis • Retinitis • Iridocyclitis • Epiescleritis
**Psychiatric**	• Delirium • Depression • Anxiety • Loss of memory

*: Seen also in children.

A common denominator of the 0.5% of patients who develop these systemic atypical patterns of disease is having some kind of predisposing condition, disease, or advanced age ^9, 16, 24^. In retrospective records of severe cases reported by Economopolou A., *et al.* from La Réunion, 89% had previous medical conditions, 78% took medication before the disease (14% NSAIDS) and 14% were alcoholic ^11, 23^. Nevertheless, it is notable that risk of severe infection and compromise seems to increase in large outbreaks, as documented in India (2006), where only 25% of cases developed classical CHIKV; and 75% were severe cases where 60% of these had some degree of neurological compromise ^10^.

### Synthesis of results

***Cardiovascular involvement.*** La Réunion reported an overall outbreak mortality of 10%; heart failure was the attributed cause in 15% of the cases, myocarditis and pericarditis in 5% and acute myocardial infarction in 2%; leaving a remarkable total of 22% mortality due to cardiovascular compromise ^11, 15^. Several similar past records raise concerns about a possible cardiac tropism of CHIKV, with clear evidence. The first description of clinical myocardial involvement of CHIKV infection was reported in 1972, when Obeyeskere *et al* presented a cohort of 10 patients who had a history of arbovirus-like syndrome, serological evidence of Dengue IgM antibodies or CHIKV haemagglutination inhibition (HI) antibodies test, and complement-fixation antibodies tst in high titres, and now had clinical and electrocardiographic evidence of myocarditis. Apart from the classic acute febrile symptoms, patients manifested palpitations, chest pain, fatigue, dyspnea and vagal-stimulation symptoms; which by themselves could already indicate coronary syndrome ^20^.

Further studies have histopathologically identified and verified the presence of the virus in cardiac tissue of postmortem biopsies. Lemant *et al* reported the case of an elderly woman with serologically confirmed CHIKV who developed a fulminant myocarditis, with no significant medical background ^29^. Myocardial biopsy revealed extensive necrosis and cytoplasmic viral inclusions in the cells ^29^. Nowadays, evidence shows that, besides the heart, CHIKV may also have tropism for the nervous system and the liver ^28^.

***Physiopathology of CHIKV-induced cardiac compromise.*** Few authors have tried to determine the physiopathology behind the cardiac damage that CHIKV can potentially cause ^19, 20^. Studying other viruses that share tropism for the heart is essential. A postmortem study, based on endomyocardial biopsies with PCR, in patients diagnosed with idiopathic dilated cardiomyopathy, evidenced a viral infiltration of myocytes in 66% of the cases. In that study the three most isolated viral agents were: parvovirus 19, herpes virus and showing that direct viral organ invasion is feasible, lethal and more frequent than expected for such viruses.

CHIKV penetrates the myocytes and generates direct damage to the muscle fibers, meanwhile inflammatory response and infiltrate grows, leading to secondary damage by a hypersensitivity reaction and necrosis, but usually with no typical signs of infarction ^20, 22, 30^. Furthermore, it has been proposed that these alterations are long-standing, and tend to make the cardiac tissue more vulnerable to recurrent damage from other microorganisms ^20^ and favor transition from myocarditis to dilated cardiomyopathy ^30^. As has been mentioned, Obeyeskere *et al* in 1972 was the first group to make such reports and observed the CHIKV physiopathology at cardiovascular level.

***Clinical cardiovascular progression pattern.*** A progression pattern has been identified and proposed, with three phases. Patients may follow the three phases strictly, or present a torpid evolution right to the last phase and skip the second one. Also, time of progression varies between individuals, depending on the severity of the initial cardiovascular injuries and previous comorbidities.

First is “pre-congestive or prodromal”; when isolated, not very specific electrocardiographic findings are detected (especially T wave abnormalities). Cardiomegaly can be detected with a simple thorax radiography or echocardiogram and gallop rhythm may be auscultated, but there are no visible cardiovascular symptoms. By this time (after 7 days), the initial viremia peak is over, but we are in front of an incipient heart failure ^19^.

The most documented electrocardiographic changes were T wave inversion in DII, III, aVF and V5-V6, and ST elevation ^18, 23, 28, 29^. These are relatively nonspecific findings, which are encouraged to be interpreted within the whole clinical context so that other compatible differential diagnoses such as acute coronary syndrome, electrolyte disorder, or even digitalis intoxication, can be dismissed ^20^. In addition, echocardiograms mostly reveal biventricular hypertrophy and dyskinesia of wall movements; and these results are compatible with myocarditis. Ejection fraction may be mildly diminished and pericardial effusion is rare. Creatine Phospho-Kinase (CPK) levels may be increased after the first phase ^28^.

The second phase is known as the “arrhythmic phase”. It starts when the recent myocardial injury can no longer permit an adequate functioning of the cardiac conduction system. Again, according to the severity, findings may range from premature auricular and ventricular extrasystoles to atrial fibrillation with high risk of thromboembolism; and in the worst-case scenario, ventricular fibrillation and sudden death ^19^. This wide spectrum directly correlates with the symptoms and hemodynamic state of the patient ^31^.

The patients after the acute and subacute phase that are most affected will invariably develop heart failure, displaying some a right side insufficiency with pulmonary and peripheral edema and hepatomegaly; but more frequently a left side insufficiency with low perfusion and shock clinic ^19^. Reduced peripheral blood flow can be responsible for many pathological events too, blurring the line between expected consequences of shock and the real direct organ damage of CHIKV. Kidneys are an example, as in Economopolou *et al’s* retrospective study, 20% of the patients with heart failure also presented with pre-renal failure ^23^, which suggests it is more of a consequence of shock in this instance. In contrast, lesions such as nephritis are more likely to be caused by the virus. Additionally, in this third stage, a constrictive syndrome has also been described, with extensive compromise and pericarditis, but it is indeed less common ^24^.

A summary of most the common clinical manifestations during CHIKV infection that suggest cardiac viral compromise is given in Table 3. Isolated signs and symptoms reported in single case reports that seemed to relate more to the pre-morbidities of the patient were excluded. Regarding blood pressure, there are significant variations in the reports, but recently hypotension was reported during acute CHIKV infection in patients with high blood pressure undergoing antihypertensive treatment. A pattern through the revised articles could not be identified, so having hypo or hypertension may be a poor predictor of cardiac compromise during CHIKV infection and seems more a product of the severity of the case and the numbers previously managed by the patient ^20^.

**Table 3.** Key clinical findings during CHIKV infection that suggest cardiac viral compromise ^12, 19– 22^. AV: Atrioventricular. NTproBNP: N-terminal pro-Brain Natriuretic Peptide. MRI: Magnetic Resonance Imaging.

**Scenario**	**Cardiovascular clinical findings**
**Data of symptoms in** **medical record**	• Chest pain, more specifically substernal • Fatigue • Dyspnea • Palpitations • Intolerance to exercise • Vagal symptoms: diaphoresis, paleness, cough, nausea, lypotimia/syncope, dizziness • Maleolar edema
**Physical examination**	• Tachycardia • Atrial and ventricular premature ectopic beats • Crepitation or rhonchi in pulmonary basis • Murmurs/third heart sound • Gallop rhythm • Irregular pulse • Jugular ingurgitation and raised jugular pressure (positive hepatojugular reflex) • Tachypnea
**Electrocardiography**	• Tachycardia • T wave inversion in II, III, aVF and V5-V5 leads • Prolonged ST segment • Deep S in V2 • Prominent R in V5 • Extrasystoles • AV blocks • Atrial fibrillation
**Laboratory**	Besides serologic IgM confirmation of CHIKV: • Increased troponins • High NTproNBP
**Radiography**	• Augmented cardio-thoracic ratio • Pleural effusion
**Echocardiogram**	• Diffuse hypokinesia, asynergia of wall movements • Ventricular hypertrophy (mostly left) • Dilated chambers • Ventricular ejection fraction may be preserved
**Contrast-enhanced** **MRI**	• Intramyocardial and subepicardial foci with increased signal intensities suggestive of necrosis (not corresponding to coronary vascular expected distribution)

***Diagnosis and management of CHIKV infection with cardiac compromise.*** Diagnosis of a CHIKV infection with cardiac compromise must be more epidemiological and clinical based rather than anything else. Specific CHIKV infection during acute phase could be diagnosed by molecular techniques such as PCR, but the phases that follow must be diagnosed by immunological/serological tests, particularly the detection of IgG anti-CHIKV. Once CHIKV infection is suspected, echocardiographic imaging, MRI and other paraclinical exams will only help in assessing the severity of the damage. There is an evident lack of studies on the topic and therefore, lack of data determining sensibility and specificity of the findings that are mentioned in Table 3. However, Simon *et al*. mentioned and delimited specific and very valid diagnostic criteria for what is called CHIKV-induced myopericarditis in their case report. They demonstrated clinical, biological and morphological evidence of myocarditis, with serologically documented CHIKV infection and no serological evidence of another recent infection, then linked the cardiovascular compromise to CHIKV ^18, 28^. Results like these are very useful but it is always advisable to always look at these criteria in the context of the patients and their previous comorbidities.

Nevertheless, what is noticed is that diagnoses are rarely made, interventions tend to be delayed and insufficient, and outcome is often an imminent refractory heart failure. Management has mostly been ineffective in containing the damage, and death by cardiac arrest becomes inevitable. Cases as severe as a 63-year-old woman with a T wave inversion in V5–V6 and global progressive hypokinesia have been reported. She experienced cardiac arrest and died 4 hours after admission ^25^, where action time was so limited and management was not even mentioned ^29^. It is not possible to state the standard management process for cardiac compromise from CHIKV infection due to the low frequency of reports of this type of CHIKV disease, so the only possibility available is to analyse management given to past reported cases in the literature and compare outcomes.

On the other hand, the treatment given to a successful case in India who remained fully asymptomatic after follow ups consisted of inotropic support (dopamine and dobutamine) and levocarnitine used to relieve mitochondrial dysfunction. Additionally, a 19-year-old male who was previously healthy developed myocarditis but was discharged after 3 days with Acebutolol and Ramipril, and at follow-up, premature beats had disappeared ^22^. There is another case of a 21-year old woman who returned from La Reunion and responded clinically to with high doses of aspirin, and her EKG changes reverted ^18^. Such good prognosis as seen in the aforementioned cases may not be representative of the true clinical progression, and may be biased due to the early age of the patients ^28^.

In summary, management of CHIKV disease is not established everywhere, remains very variable, and consists mainly in correcting the clinical features of the cardiac failure, but does not taking into consideration the root cause. Beta-adrenergic blockers, ACE-inhibitors and inotropic support during the crisis are commonly reported in order to maintain hemodynamic stability. Only one case reported the use of prednisolone ^21^, but without any other cardiac support drugs, and the outcome was equally poor. Studies on the impact of anti-inflammatory corticosteroids along with cardiovascular support drugs should be carried out, it seems to be a promising option considering the underlying severe systemic inflammatory response in these cases. A very similar substrate is seen in the eosinophilic myocarditis that can cause *Toxocara canis*, where early prednisolone in doses of 1mg/kg/day for the acute phase and 5–10mg/kg/day for maintenance has been recommended ^32^.

***Prognosis and functional sequelae.*** The Indian child cited above showed general improvement within three days, with no relapses. A follow up echocardiogram reported only a mild mitral regurgitation, with intact left ventricle function ^28^. The 19 and 21 year old patients remained asymptomatic, but dilation persisted on imaging ^18, 21^. By now, it is evident that there are three clear, different outcomes to CHIKV infection:

• Asymptomatic with no imaging sequelae;
• Asymptomatic with partial reversion of EKG and echocardiogram changes;
• Death

Changes seen on cardiac magnetic resonance imaging that persist for more than one year from disease onset will be permanent and affect the patients to some degree later in life ^18^. Simon *et al* thus proposes that in upcoming years, countries that suffered outbreaks of CHIKV since 2005, will see a long-term increase in dilated cardiomyopathy, reporting this as the most frequent sequelae, even in asymptomatic patients who had an apparently classic clinical picture involving arthralgia predominantly ^18^. This raises public health concerns and the risk of a noticeable limitation in quality of life for these patients in the future.

**Similar reports and findings on Dengue fever: Arbovirus-induced cardiopathy.** The cardiac tropism of CHIKV seems to be shared with DENV, with multiple cases in the literature displaying similar cardiovascular complications and often mimicking acute myocardial infarction as well ^33, 34^. Myocarditis is reported similarly. However, arrhythmias and compromise of the electric conduction system of the heart have a higher incidence with DENV, including supraventricular arrhythmias such as atrial fibrillation, atrioventricular (AV) blockage ^28^ and cases reporting refractory ventricular fibrillation as the ultimate cause of death ^34^. Acute pericardial and pulmonary edema are also described, but the outcome is rarely fatal. As a common denominator in the published literature, most reports of cardiac involvement are seen in patients with hemorrhagic fever manifestations of CHIKV infection.

### Physiopathology

Even though the etiological agent is very similar, DENV-induced cardiomyopathy has a variant: the plasma leak syndrome and characteristically endothelial dysfunction of DENV that may result helpful to the extravasation process and chemotaxis of inflammatory cells to myocardial tissue, creating a highly cytokine rich environment ^35^, besides the already known tropism of DENV for the heart. This could explain why cardiovascular manifestations are much more common with DENV than with CHIKV ^35, 36^. Host susceptibility and the virulence of the strain also play a role in the severity of the clinical picture ^37^.

### Electrocardiography and echocardiography

Manifestations remain comparable, but electrocardiography disturbances are observed frequently in a wide range of 34–75% ^35, 36^ of the dengue cases. In the 2005 outbreak, Sri Lanka reported 62.5% of patients affected ^37^. Abnormalities basically consist of sinus bradycardia, T inversion, depression of ST segment in precordial leads and avF, AV blocks, (Mobitz type I second degree has been mentioned) bundle branch blocks and rarely, atrial fibrillation ^33, 34, 37, 38^. All were reported as supposedly transient ^39^. Two cases of remaining atrial fibrillation after the resolution of disease have been reported, with reversion only achieved after antiarrhythmic treatment (Amiodarone) ^39^.

Imaging is similar to what is reported in CHIKV echocardiography: global hypokinesia and important decrease in left ventricle ejection fraction (LVEF). A study reported a mean of 47.08% of LVEF in all DENV infected patients, and of 39.6% if shock syndrome was present. At follow up after three weeks, LVEF was superior to 50% in all cases and ECG changes had reverted ^35^. From these findings, JP Wali *et al* proposed three diagnostic criteria for suspected cardiac compromise: ST-T changes in ECG, global hypokinesia and a decreased LVEF in imaging.

Although arboviral cardiovascular manifestations have been described for over 40 years ^20^, few studies ^8, 18, 40^ have documented in detail the specific cardiovascular and specific EKG patterns during acute disease ^40^, especially in recent epidemics in Latin America. Initial reports of three fatal cases of chikungunya in Barranquilla, Colombia ^15^, in which patients presented hypotension and tachycardia, raised red flags among physicians in the region. More recently in Sucre, Colombia, in 2016, a case series of 42 patients with chikungunya followed in detail found arrhythmias in EKG findings, such as repolarization disturbances, in more than 71% of those cases. Repolarization disturbances were the most frequent (21%) ^40^. Preliminary unpublished data ^41^ from a study in Caracas, Venezuela, reported in 2016, they provided similar findings in patients, although at a lower frequency. Indeed, evidence of patent or silent myocarditis was observed in a high percentage of patients prospectively evaluated in Venezuela. An unexpected finding was persistent symptomatic arterial hypotension observed in one third of these patients with prior stable hypertension on treatment, requiring the anti-hypertensive medication to be discontinued or reduced due to severe clinical manifestations ^41^.

A study from Tolima, Colombia, carried out in 2016 provided consistent findings and information with regards to the spectrum of EKG alterations. Rhythm disturbances occurred in 10 patients out of 14 (71%) ^35^. They included sinusal tachycardia (3/14 patients), hemiblocks (2/14), left ventricular hypertrophy (2/14) and ST segment depression (2/14), among others ^35^.

Patients with chikungunya may present cardiovascular complications including myocarditis and pericarditis ^18, 40, 41^. Thus, an accurate physical examination, including a detailed cardiovascular system assessment should be performed. This should include cardiac auscultation looking for sound alterations, which could be indicating premature ventricular contractions ^18, 20, 40, 41^. Besides that, all CHIKV infected patients with should have an EKG performed on them, given that it is an easy, cheap and quick assessment tool that could prevent potential deleterious cardiovascular outcomes ^40^.

In light of any clinical or electrocardiographic abnormality, cardiac enzymes should also be measured (e.g. troponin) ^20^. As suggested for over 40 years ^20^ cardiac tropism and direct cytolytic effects of the virus remains a latent possibility ^40^, yet to date has not been demonstrated at a tissue level. Further studies using novel molecular approaches for virus detection in endomyocardial biopsies of symptomatic CHIKV infected patients could confirm this possible role and establish the underlying physiopathological mechanisms of CHIKV myocarditis which then translate into the the spectrum of symptoms such as rhythm and conduction disturbances ^20, 40^.

Ongoing studies should focus on determining the potential chronic cardiovascular outcomes that could develop in patients infected with chikungunya, in order to provide an appropriate early clinical intervention strategy to avoid potential disabilities.

### Management

Management of DENV is poorly reported and not established everywhere, as is the case with CHIKV. Early use of IV hydrocortisone resulted in full recovery in two cases of myocarditis in 12 year old patients ^42^, and authors support that fatality is significantly reduced under opportune intervention during the first hours ^42^. A more conservative attitude was adopted for the analyzed cohort from the Sri Lanka outbreak; with indications of strict bed rest, liquid maintenance, oxygen, close monitorization of vital signs and inotropic support when needed, and a clear avoidance of steroids and other empirical drugs ^37^.

The importance of a rapid intervention (first hours) is exemplified by the case of a 25 year old Indian male, that presented with nonspecific abdominal epigastric pain and vomiting. Exams revealed myocarditis. The patient died in a few hours when he developed pharmacological and electrical refractory ventricular tachycardia while evaluating a much more invasive treatment option: the possibility of implanting a left ventricular assistance device. Positive DENV serology results were known later ^34^. It is clear at this point, that therapy needs to be standardized for arbovirus-induced cardiomyopathy, comparing efficacy of treatments that have already been proposed, as well as new treatment options.

## Discussion

The key for a successful outcome of CHIKV-induced cardiomyopathy is recognizing signs and symptoms early on. It is certainly a condition that can be life-threatening, which is why patients should be referred for cardiac assessment as early as possible, after displaying any of the previously mentioned symptoms. Identifying comorbidities is recommended as well to distinguish CHIKV-induced cardiomyopathy from an exacerbation of previous heart disease.

For dengue virus infection, it is now known that the cardiovascular involvement is mostly characterized by rhythm abnormalities (bradycardia), with no symptoms or complications. However, in moderate or severe cases where there was a cardiovascular affectation or complication, myocarditis has been an important issue. Myocarditis due to DENV infection may present several patterns such as “refractory shock”, “heart failure”, “arrhythmia”, etc and It would be important to consider this diagnosis. In the case of CHIKV infection and cardiac involvement, myocarditis should be also considered.

Cardiac compromise is not so common in isolated episodes; but countries where chikungunya virus is an epidemic should be alarmed and well informed about this condition. Physicians should be encouraged to keep divulgating reports on the cardiovascular involvement of chikungunya virus disease, to raise awareness and ultimately encourage suitable diagnosis and intervention worldwide. Questions are still raised about the real incidence, as every outbreak seems to follow a different pattern, but what is needed the most is further investigation on therapy for this specific condition and in different age groups.

A significant issue arises with the diagnosis of myocarditis by arboviruses such as DENV and CHIKV, because a myocardial biopsy or cardiac magnetic resonance imaging needs to be considered and performed. However, performing these, in tropical areas where these arboviruses are prevalent, is very hard and there are many restrictions. The management of myocarditis, regardless its etiology, should be focused in the therapeutics oriented to the agent (virus, bacteria, etc); the cardiovascular events support (controllling heart failure, cardiogenic shock, arrhythmia, etc) and the treatment of the inflammatory process. The last one is under discussion and needs more research, although in some severe cases due to DENV, the corticosteroids administration changed the evolution given their positive benefits.

CHIKV would induce not just cardiovascular compromise and cardiovascular manifestations during the acute phase, but also at subacute and chronic stages 43–56. Today it is known that compromise during chronic disease is not just limited to the rheumatological manifestations. Nevertheless, in CHIKV, the definition of systemic manifestation or extra-articular compromise has not well defined. But in the case of atypical conditions, this was defined by PAHO/WHO during the expert consultation meeting in Managua, Nicaragua, 2016, and later published by WHO ^43^. 

### Limitations

Limitations will always be the sporadic nature of these cases, something we need to be prepared for in future outbreaks. Methodologically, it should also be considered that the inclusion of reviews may cause a bias, but in some cases, some would serve to locate some additional key articles useful for a novel topic such as the one of this systematic review. Additionally, many studies did not report detailed information about laboratory diagnoses (Troponin, BNP, CK-MB, etc), imaging studies (echocardiography, magnetic resonance), final diagnoses (myocarditis, etc), as well management (inotropics, corticosteroids, etc) and outcomes (survival, death). Additionally, articles published in French have been excluded. This could be a limitation as the largest series of cases and their complications came from France in relation to the outbreak on La Reunion Island, and many of these were published in French. Nevertheless, from these publications we did not identify, initially by the title or the abstract, any relating to cardiovascular complications.

## Conclusions

Finally, these observations on DENV and CHIKV associated cardiovascular manifestations could be useful for management of Zika virus infections, which are currently causing epidemics in Latin America ^44– 46^. Cardiovascular compromise has already been described and reported in fatal cases ^47, 48^. In addition, cardiovascular complications might be underdiagnosed in clinical practice ^49^. Future research needs to focus on the potential cardiovascular complications of Zika virus infection, with prompt cardiovascular screening in suspected cases ^45, 49, 50^. Other emerging arboviruses such as Mayaro ^50– 55^, Oropouche ^52, 53^, Venezuelan Equine Encephalitis ^54, 55^ may be also causing cardiovascular compromise, or even be co-infecting. We are still learning about the multiple clinical implications ^56, 57^ of co-infection, including those affecting the cardiovascular system.

## Supplementary material

Supplementary file 1: PRISMA checklist.

.

Supplementary file 2: PRISMA flowchart showing the number of records identified, included and excluded.
