# Supplementary material for: Cardiovascular involvement and manifestations of systemic Chikungunya virus infection: A systematic review
Source: F1000Res. 2017 May 2;6:390. Originally published 2017 Mar 29. [Version 2] doi: 10.12688/f1000research.11078.2 (PMC5405794; doi:10.12688/f1000research.11078.2)
Supplement: Supplementary file 2 [file f1000research-6-12414-s0001.tgz › d8c275ca-d68b-4647-8a53-4ad007d03a9a.docx]

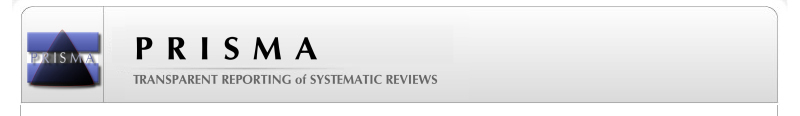
**PRISMA 2009 Flow Diagram**

Studies included in quantitative synthesis (meta-analysis)
(n = 0)

Studies included in qualitative synthesis
(n = 40)

Full-text articles excluded, with reasons
(n = 30)

Full-text articles assessed for eligibility
(n = 70)

Records excluded
(n = 620)

Records screened
(n = 690)

Records after duplicates removed
(n = 690)

Additional records identified through other sources
(n = 0)

## Identification

## Eligibility

## Included

## Screening

Records identified through database searching
(n = 737)
