# Supplementary material for: Cardiovascular involvement and manifestations of systemic Chikungunya virus infection: A systematic review
Source: F1000Res. 2017 May 2;6:390. Originally published 2017 Mar 29. [Version 2] doi: 10.12688/f1000research.11078.2 (PMC5405794; doi:10.12688/f1000research.11078.2)
Supplement: Peer review history

# Peer review: Referee response for version 1

**DOI:** 10.5256/f1000research.11949.r21386

**Published:** 2017-04-25

**Authors:** Cecilia Perret

**Affiliations:** 1 División de Pediatría, Departamento de Enfermedades Infecciosas e Inmunología Pediátricas, Escuela de Medicina, Pontificia Universidad Católica de Chile, Santiago, Chile

General comment

 This is a very interesting article that compiles information relevant to an emerging and widely disseminated infection in the American continent whose complications are still under study and its impact still to be known.

 Specifically, there are some aspects of the review that need to be considered:

 Objectives:

 - Clearly specified focusing on the cardiovascular involvement of Chikungunya virus infection, frequency of presentation, clinical manifestations and laboratory elements such as the electrocardiogram

 Methodology

 - Articles published in French have been left out, which could be a limitation considering that the largest series of cases and their complications came from France in relation to the big outbreak on La Reunion island, and many of them published in French.

 - The search criteria are wide: Chikungunya AND systemic manifestations, heart, cardiac. However, with these criteria, you could lose some reports of severe disease that do not appear under these search criteria. Chikungunya AND mortality or Death could be included. Also, more specific criteria could be used in order to answer the question the authors propose to find cardiovascular involvement in Chikungunya infection

 - The inclusion of dengue in the search and in the results escapes the objectives of this systematic review.

 There is no reference to the use of PRISMA checklist in this review, if it was used, make it more explicit in each of the items.

 Results

- It is confusing and difficult to follow what results are obtained from the systematic review (40 articles) and what results are from articles not included in the review.
- Table 1, which shows the frequency of the involvement of different organs in chikungunya can be absolutely biased since the inclusion criteria are articles with systemic involvement. According to the search criteria, the classical form of the disease has not been included overestimating the frequency of systemic involvement.
- It is not clear what criteria you used to classify an organ involvement as very common or extremely rare. According to the authors, an unusual manifestation occurs between 39-20%. This could be debatable.
- The clinical description of the disease in terms of acute, post-acute and chronic phase is irrelevant for the purposes of this review. Just mention the clinical aspects that are important for the objectives. In this sense, clarify the terms used as atypical manifestations, extra-articular manifestations, systemic disease, severe disease. They are used sometimes as synonyms and sometimes with a different meaning. It is confusing. It is difficult to understand why in table 1, cardiovascular manifestations are as frequent as 54% but does not appear to be so in the text of atypical presentations.
- The entire section of cardiovascular manifestations in dengue goes beyond the purpose of the study and should not be mentioned in the results. Comparisons with the cardiovascular compromise in chikungunya, which is appreciated, can be presented in the discussion.
- At the end of results and before management, the paragraph of studies determining the cardiovascular outcome in patients with chikungunya should be included in conclusions.

 

 Discussion

 It is not clear the meaning of the phrase cardiac compromise is not so common in isolated episodes.

 The authors do not clearly indicate the home take messages for this study and its main contribution.

 Limitations:  authors do not mention the limitations of their study.

 Conclusions: Do not correspond to purpose of the study or objectives.

 References

 In the references, it is not clear what are the articles included in the systematic review and what are included for discussion.

I have read this submission. I believe that I have an appropriate level of expertise to confirm that it is of an acceptable scientific standard, however I have significant reservations, as outlined above.

# Response

**Published:** 2017-04-26

**Authors:** Alfonso Rodriguez-Morales

**Affiliations:** Universidad Tecnológica de Pereira, Colombia

Dear Dr. Perret

Thanks for your valuable comments. Certainly the topic of this systematic review (without meta-analysis), is on ongoing findings that in the near future will be better defined. Regard your assessment, unfortunately this arrived when two others reviews ago had been submitted; based on them, we have recently proceeded to develop the new revised version. Your comments are consistent with those from Dr. Vilcarromero in a significant magnitude. Then, most of your comments are addressed in the revised version of the paper, including clarification on the objectives as well on the Methodology (explaining more the search criteria as well the clarification regard the use of PRISMA checklist and flow diagram). Regard the language of articles included, we agree that this would be a limitation. Limitations are now better described in the new version of the article. As we explained, dengue cardiovascular compromise and manifestations was a necessary comparison for understanding of readers. In the new version, Discussion and Conclusions were improved.

# Peer review: Referee response for version 1

**DOI:** 10.5256/f1000research.11949.r21382

**Published:** 2017-04-18

**Authors:** Stalin Vilcarromero

**Affiliations:** 1 Sociedad Científica de Estudiantes de Medicina de la Amazonía Peruana (SOCIEMAP), Facultad de Medicina, Universidad Nacional de la Amazonía Peruana, Iquitos, Peru; 2 Naval Medical Research Unit № 6, Lima & Iquitos, Peru

In the manuscript entitled “Cardiovascular involvement and manifestations of systemic Chikungunya virus infection: A systematic review” authors have made a hard and interesting description of the cardiovascular effect by chikungunya virus infection. This analysis would be important to share with the scientific community; however it is necessary major changes in order to be ready for publication.

 Mainly, focus in the specific topic and put in order and properly the information in the paper.

**General comments:**

1. Objective:

It s not clear, author start assessing that the objective focus among cardiovascular involvement and manifestation, however, other systemic manifestation or atypical manifestation also become relevant in the results section.

1. Methodology

1. The main objective of systematic reviews is to respond to a specific question. Initially authors seem to do that pointing on cardiovascular involvement, but then, when they write the introduction and results section, this main point seem to disappear or is not clear for the reader. However, in the discussion section, authors re-take the objectives again. Certainly, the big amount of information given in the introduction section and talking about the virus, history, classification, vector and cycles is very interesting, but it is not the purpose of this study. I suggest them shortening it and focussing on the cardiovascular involvement and complications. I also recommend displaying a flow (figure) of how papers were excluded and included.
2. What guideline do the authors follow up in order to assess the risk of bias (PRISMA or Cochrane? It is possible you follow PRISMA approach (For example: http://prisma-statement.org/PRISMAStatement/Checklist.aspx). Please give this information explaining the steps.
3. In the Methods section, the next key word “Chikungunya AND Systemic AND Manifestation” used by authors has probably given them no specific references or papers. Instead of that, why do not they also use specific “key words” such as: “Chikungunya AND cardiac involvement”  “Chikungunya AND cardiac complication” or “Chikungunya AND cardiovascular involvement”  “Chikungunya AND cardiovascular complications” or Chikungunya AND Atypical manifestation/complications“. The idea is to be more specific and less general.
4. According to the authors, the protocol was registered in PROSPERO; however it was not possible to view the registered protocol in the web (https://www.crd.york.ac.uk/prospero/searchadvanced.php).  
5. I wonder if the inclusion of reviews may cause a bias. Please clarify and if it is true, consider in the “limitations section”.

1. Results
  1. Authors use “cardiac affectation” and also “cardiovascular compromise”. I recommend to standardize the term in order to avoid creating confusion for the reader.
  2. Authors describe with detail the clinical features during the acute, post-acute and chronic stage of CHIKV infection. I recommend shortening and focusing on the topic.
  3. In “Atypical presentation” section, authors shows systemic manifestation considering the affectation in different organs such as neurological, cardiovascular, etc.  Why did not the authors use also the key word “atypical” rather than “systemic manifestation” or “extra-articular” in the searching strategy and also in the analyses? It is confusing.
  4. In Table 2, the word “Systemic extra-articular involvement of atypical CHIKV” shows clinical manifestation, however, we do not know more information about “who (co-morbidities? Older? Young?)”, “how many”(number/percentage), type of paper(case report, case control, etc), etc. Are these clinical manifestations in outpatients or inpatients? Are these early clinical features or complications? So there are many questions around this information that would be useful in order to form a correct interpretation.
  5. In Table 3, it is important to consider more information about the selected papers. I recommend a big table showing the different cardiovascular involvement/complication, for example: 1. the clinical diagnoses (heart failure, acute coronary syndrome, refractory shock, and rhythm abnormalities), relevant signs and symptoms, especially early signs/symptoms (chest pain, dyspnea, bradycardia, etc), laboratory diagnose (Troponin, BNP, CK-MB, etc), Imaging studies (echocardiography, Magnetic resonance), final diagnose (Myocarditis?), Management (inotropics, corticosteroid, etc) and outcome (survive, died). I consider, this data would be important to understand the impact of cardiovascular involvement due to CHIKV.
  6. In dengue, now it is known that more about the cardiovascular involvement is mostly characterized by rhythm abnormalities (bradycardia) with no symptoms or complications. However, in moderate or severe cases where there was a cardiovascular affectation or complication, myocarditis has been an important cause. Myocarditis due to DENV infection may present several patterns such as “refractory shock”, “heart failure”, “arrhythmia”, etc and It would be important to consider this diagnose. In the comparison with CHIKV infection and cardiac involvement, myocarditis, should be discussed.  It is not clear what do authors try to assess when they say: “The cardiac tropism of CHIKV seem to be shared with DENV”, it is important to clarify.
  7. Diagnose myocarditis by those arbovirus, need to consider a myocardial biopsy or a cardiac magnetic resonance, however performing these in tropical areas where these arbovirus are prevalent, is very hard.
  8. The management of myocarditis, whatever is the etiology, focus in the management of the agent (virus, bacteria, etc); management of the cardiovascular event (heart failure, cardiogénico shock, arrhythmia, etc) and the management of the inflammatory process. The last, is under discussion and needs more research, although in some severe cases by DENV, the corticosteroids administration changed the evolution.
2. Discussion

Authors say, “The key for a successful outcome of CHIKV-induced cardiomyopathy is recognizing signs and symptoms”. Here I recommend repeating the most important ones.

1. Limitations

This section would consider some of the bias common to this kind of study where there is no clinical trials included and where “case reports” were including. Bias such as “selection” or

 “publication” bias, etc.

I have read this submission. I believe that I have an appropriate level of expertise to confirm that it is of an acceptable scientific standard, however I have significant reservations, as outlined above.

# Response

**Published:** 2017-04-19

**Authors:** Alfonso Rodriguez-Morales

**Affiliations:** Universidad Tecnológica de Pereira, Colombia

Dear Dr. Vilcarromero

First, thanks for your initial comments on our systematic review (SR) (without meta-analysis). In second place we want to clarify that making for the first time a SR about cardiovascular involvement and manifestations of systemic Chikungunya virus infection implies comparisons with dengue, unavoidably, as well, to introduce readers about the systemic manifestations of chikungunya, to better understand the cardiovascular involvement and manifestations in this arboviral disease. By the way, still on this specific topic, many aspects will become more detailed along with research in the near future. In which our group is specifically also contributing with studies, already published on the cardiovascular electrocardiographical alterations, as well ongoing on the ecochardiographical and cardiovascular biochemical ones.

**Objective:**

**It s not clear, author start assessing that the objective focus among cardiovascular involvement and manifestation, however, other systemic manifestation or atypical manifestation also become relevant in the results section.**

Our SR has as objectives, not just one:

-To systematically review published literature on the cardiovascular manifestations and involvement of systemic CHIKV infection;

-To explore which are the main clinical cardiovascular features of chikungunya infection?

-To identify which are the main electrocardiographical findings of chikungunya infection?

All of them, are clearly developed in the section Synthesis of Results, which is the main part of the SR.

Before that section, a brief introduction on other systemic manifestation or atypical manifestation of chikungunya infection is at Clinical Course, short section of just 3 paragraphs and atypical presentations, a section of just 2 paragraphs, which include, by the way, the transition to the cardiovascular aspects, developed in the section Synthesis of Results and later sections of the SR.

**Methodology**

**The main objective of systematic reviews is to respond to a specific question.**

In our case, to three specific questions, with the proper context to make it readable and understandable given the novelty of the topic, as mentioned, also with ongoing research in multiple aspects.

**Initially authors seem to do that pointing on cardiovascular involvement, but then, when they write the introduction and results section, this main point seem to disappear or is not clear for the reader.**

From the title it's clear that this SR is not only about the cardiovascular involvement, but also about cardiovascular manifestations of chikungunya. Nevertheless, we will make more clarifications on our Introduction regarding this.

**However, in the discussion section, authors re-take the objectives again.**

Ok. Thanks.

**Certainly, the big amount of information given in the introduction section and talking about the virus, history, classification, vector and cycles is very interesting, but it is not the purpose of this study.**

This is not properly a study, it is a SR, without meta-analysis. Then, we consider, given the still novelty of the topic, such aspects are necessary for the readers.

**I suggest them shortening it and focussing on the cardiovascular involvement and complications.**

We have done that at Synthesis of Results and later sections of the SR.

**I also recommend displaying a flow (figure) of how papers were excluded and included.**

With our submission we provided the PRISMA Flow Chart and Checklist. However, this was not published by the journal with our article. Considering its importance and agreeing with you about it, we will incorporate as a Figure 1 and including properly in the manuscript, and not as supplementary file of our submission.

**What guideline do the authors follow up in order to assess the risk of bias (PRISMA or Cochrane? It is possible you follow PRISMA approach (For example: http://prisma-statement.org/PRISMAStatement/Checklist.aspx). Please give this information explaining the steps.**

We followed the PRISMA statement, as recommended by F1000 Research. We will clarify more on this in our revised manuscript.

**In the Methods section, the next key word “Chikungunya AND Systemic AND Manifestation” used by authors has probably given them no specific references or papers. Instead of that, why do not they also use specific “key words” such as: “Chikungunya AND cardiac involvement”  “Chikungunya AND cardiac complication” or “Chikungunya AND cardiovascular involvement”  “Chikungunya AND cardiovascular complications” or Chikungunya AND Atypical manifestation/complications“. The idea is to be more specific and less general.**

Given the lack of studies, we explore both options, finally trying to be more sensitive in order to include all the possibly relevant studies related to our SR.

**According to the authors, the protocol was registered in PROSPERO; however it was not possible to view the registered protocol in the web (https://www.crd.york.ac.uk/prospero/searchadvanced.php). ** 

We agree with you. This protocol was not prospectively registered in PROSPERO. But this is not a mandatory aspect for publication at F1000 Research, then this was modified later, but when published appeared with that incorrect comment. This will be deleted in the revised version.

**I wonder if the inclusion of reviews may cause a bias. Please clarify and if it is true, consider in the “limitations section”.**

We will extend more on our Limitations.

**Results**

**Authors use “cardiac affectation” and also “cardiovascular compromise”. I recommend to standardize the term in order to avoid creating confusion for the reader.**

Agree, we will use only "compromise" and not "affectation".

**Authors describe with detail the clinical features during the acute, post-acute and chronic stage of CHIKV infection. I recommend shortening and focusing on the topic.**

We will consider this in the revised version.

**In “Atypical presentation” section, authors shows systemic manifestation considering the affectation in different organs such as neurological, cardiovascular, etc.  Why did not the authors use also the key word “atypical” rather than “systemic manifestation” or “extra-articular” in the searching strategy and also in the analyses? It is confusing.**

In chikungunya, the definition of systemic manifestation or extra-articular has not well typified. Conversely, atypical case was defined by PAHO/WHO during the expert consultation meeting in Managua, Nicaragua, 2016, and later published at the Weekly Epidemiological Record of the WHO 14 august 2015, 90, 33, 409-420. We will clarify this in our revised version.

**In Table 2, the word “Systemic extra-articular involvement of atypical CHIKV” shows clinical manifestation, however, we do not know more information about “who (co-morbidities? Older? Young?)”, “how many”(number/percentage), type of paper(case report, case control, etc), etc. Are these clinical manifestations in outpatients or inpatients? Are these early clinical features or complications? So there are many questions around this information that would be useful in order to form a correct interpretation.**

We will consider the improvement of this Table.

**In Table 3, it is important to consider more information about the selected papers. I recommend a big table showing the different cardiovascular involvement/complication, for example: 1. the clinical diagnoses (heart failure, acute coronary syndrome, refractory shock, and rhythm abnormalities), relevant signs and symptoms, especially early signs/symptoms (chest pain, dyspnea, bradycardia, etc), laboratory diagnose (Troponin, BNP, CK-MB, etc), Imaging studies (echocardiography, Magnetic resonance), final diagnose (Myocarditis?), Management (inotropics, corticosteroid, etc) and outcome (survive, died). I consider, this data would be important to understand the impact of cardiovascular involvement due to CHIKV.**

Unfortunately the number of papers as well the data available is limited to fully performed that, although was discussed by the group of authors of this SR. Nevertheless, we will consider your comment to improve this Table.

**In dengue, now it is known that more about the cardiovascular involvement is mostly characterized by rhythm abnormalities (bradycardia) with no symptoms or complications. However, in moderate or severe cases where there was a cardiovascular affectation or complication, myocarditis has been an important cause. Myocarditis due to DENV infection may present several patterns such as “refractory shock”, “heart failure”, “arrhythmia”, etc and It would be important to consider this diagnose. In the comparison with CHIKV infection and cardiac involvement, myocarditis, should be discussed.  It is not clear what do authors try to assess when they say: “The cardiac tropism of CHIKV seem to be shared with DENV”, it is important to clarify.**

Thanks for this comment. We will use it in the revised version.

**Diagnose myocarditis by those arbovirus, need to consider a myocardial biopsy or a cardiac magnetic resonance, however performing these in tropical areas where these arbovirus are prevalent, is very hard.**

We fully agree with this.

**The management of myocarditis, whatever is the etiology, focus in the management of the agent (virus, bacteria, etc); management of the cardiovascular event (heart failure, cardiogénico shock, arrhythmia, etc) and the management of the inflammatory process. The last, is under discussion and needs more research, although in some severe cases by DENV, the corticosteroids administration changed the evolution.**

We fully agree with this.

**Discussion**

**Authors say, “The key for a successful outcome of CHIKV-induced cardiomyopathy is recognizing signs and symptoms”. Here I recommend repeating the most important ones.**

Ok. Agree.

**Limitations**

**This section would consider some of the bias common to this kind of study where there is no clinical trials included and where “case reports” were including. Bias such as “selection” or “publication” bias, etc.**

We will comment more on that.

# Peer review: Referee response for version 1

**DOI:** 10.5256/f1000research.11949.r21381

**Published:** 2017-04-18

**Authors:** José Antonio Suárez

**Affiliations:** 1 Unidad Clínica de Enfermedades Tropicales, Instituto Conmemorativo Gorgas de Estudios de la Salud, Panama, Panama

Cardiovascular involvement in CHIKV disease has been described in several publication highlighting a possible cardiac tropism of CHIKV. This findings have shown that arboviruses like CHIKV and Dengue can share with parvovirus 19, herpes virus and enterovirus30 and other viruses, the list of the viral causes of heart damage.

 This study helps the understanding of cardiovascular manifestations and complications in all 3 stages of CHICK disease and it gives the physician the awareness of thinking in arbovirus-related diseases to make the accurate diagnosis and avoid fatalities. Once the physician thinks in CHIKV, the patient should have a cardiac assessment as early as possible, especially in countries where CHIKV is epidemic.

 From study design, methods and analysis points of view, the authors complied with all PRISMA and PROSPERO criteria for a Systematic Review showing a robust data and good conclusions.

 In Latin America where other arboviruses are co-circulating with CHIKV and Dengue, cardiovascular symptoms can be the first signal of a viral infection.

 The content of this Systematic Review can help tropical medicine and travel medicine physicians to have a better approach in the assessment of patients with some arbovirus diseases.

I have read this submission. I believe that I have an appropriate level of expertise to confirm that it is of an acceptable scientific standard.

# Response

**Published:** 2017-04-19

**Authors:** Alfonso Rodriguez-Morales

**Affiliations:** Universidad Tecnológica de Pereira, Colombia

Dear Dr. Suárez

Thanks for your assessment as well your positive comments on our review, which as can be appreciated after reviewing  major bibliographical databases, such as Scopus, PubMed and/or Web of Science, is probably the first to address specifically the cardiovascular involvement and manifestations of systemic Chikungunya virus infection.
